# Efficacy and safety of TMC278 in treatment-naïve, HIV-1-infected patients with HBV/HCV co-infection enrolled in the phase III ECHO and THRIVE trials

**DOI:** 10.1186/1758-2652-13-S4-P210

**Published:** 2010-11-08

**Authors:** M Nelson, G Amaya, N Clumeck, C Arns Da Cunha, D Jayaweera, P Junod, L Taisheng, P Tebas, M Stevens, A Buelens, S Vanveggel, K Boven

**Affiliations:** 1 Chelsea and Westminster Hospital, London, UK; 2 Centro Médico Puerta de Hierro, Zapopan, Jal, Mexico; 3 Saint-Pierre University Hospital, Brussels, Belgium; 4 Centro Médico São Francisco, Curitiba, Brazil; 5 University of Miami, Miami, FL, USA; 6 Clinique Medicale du Quartier Latin, Montreal, Canada; 7 Beijing PUMC Hospital, Beijing, China; 8 University of Pennsylvania, Philadelphia, PA, USA; 9 Tibotec BVBA, Beerse, Belgium; 10 Tibotec Inc., Titusville, NJ, USA

## Introduction

TMC278 had a high virologic response rate, non-inferior to EFV, in two Phase III double-blind trials ECHO (TMC278-C209, NCT00540449) and THRIVE (TMC278-C215, NCT00543725) in treatment-naïve HIV-infected adult patients. As the use of NNRTIs, particularly nevirapine, has been associated with hepatic-related adverse events (AEs), especially in HIV/hepatitis B (HBV) and/or hepatitis C (HCV) co-infected patients, a subgroup analysis of these events was performed on the pooled Week 48 Phase III data.

## Methods

Patients (N=1368) with alanine aminotransferase (ALT)/ aspartate aminotransferase (AST) ≤ 5x upper limit of normal received TMC278 25mg qd or EFV 600mg qd, plus TDF/FTC (ECHO) or TDF/FTC, AZT/3TC or ABC/3TC (THRIVE). HIV/HBV and/or HCV co-infection status was determined at baseline in 1335 patients by HBV surface antigen, HCV antibody and RNA testing.

## Results

At baseline, 112/1335 patients (8.4%) had evidence of HIV/HBV and/or HCV co-infection (randomised to TMC278, n=49: 7.3%; EFV, n=63: 9.5%). Table 1 summarises the outcomes.

Compared with HIV mono-infected patients, co-infected patients had more hepatic AEs (clinical and laboratory) and lower virologic responses, which were similar across treatment groups. Hepatic AEs rarely led to treatment discontinuation (TMC278: n=3 vs. EFV: n=9 patients). There were no fatal hepatic AEs.

**Table 1:** 

	*HIV/HBV and/or HCV co-infected patients*		*HIV mono-infected patients*	
	TMC278 25mg qd	EFV 600mg qd	TMC278 25mg qd	EFV 600mg qd

Efficacy (Week 48 outcomes)*	N=49	N=63	N=621	N=602

% (95% CI) with viral load <50 copies/mL, ITT-TLOVR	73.5 (60.7-86.3)	79.4 (69.1-89.6)	85.0 (82.2-87.8)	82.6 (79.5-85.6)

Mean CD4 count (95% CI)	N=48	N=63	N=621	N=602

Baseline, cells/mm^3^	230 (198-263)	246 (216-276)	262 (251-273)	274 (262-285)

Change from baseline, NC=F^†^, cells/mm^3^	+137 (100-175)	+192 (147-238)	+197 (186-209)	+173 (161-185)

Change from baseline, NC=F^†^, %	+6.6 (5.0-8.3)	+7.7 (6.4-9.0)	+8.6 (8.1-9.0)	+8.4 (7.9-8.8)

*Safety^‡^, ^§^*				

*Treatment-emergent hepatic AEs of interest, n (%)*	N=54	N=66	N=632	N=616

Any hepatic AE	15 (27.8)	17 (25.8)	23 (3.6)	28 (4.5)

Hepatobiliary disorders^Â¶^	3 (5.6)	7 (10.6)	6 (0.9)	9 (1.5)

HBV or HCV reported as an AE	3 (5.6)	5 (7.6)	-	-

Hepatic laboratory abnormalities reported as an AE	9 (16.7)	8 (12.1)	19 (3.0)	21 (3.4)

*Grade 3 to 4 hepatic laboratory abnormalities, n (%)*	N=54	N=66	N=631	N=604

ALT increased	9 (16.7)	11 (16.7)	1 (0.2)	12 (2.0)

AST increased	7 (13.0)	5 (7.6)	7 (1.1)	14 (2.3)

Hyperbilirubinaemia	0	0	4 (0.6)	1 (0.2)

ITT-TLOVR = intent-to-treat-time-to-loss of virologic response; CI=confidence interval; *Patients included in efficacy analysis were those with baseline HBV/HCV assessments; †NC=F = non completer = failure: missing values after discontinuation imputed with change = 0; Last observation carried forward otherwise; ‡Safety analyses performed using all available data, including beyond Week 48; §Patients who seroconverted for HBV/HCV during the study were included in the subgroup of HIV/HBV and/or HCV co-infected patients; Â¶Selection of preferred terms from System Organ Class as defined by MedDRA. Compared with HIV mono-infected patients, co-infected patients had more hepatic AEs (clinical and laboratory) and lower virologic responses, which were similar across treatment groups. Hepatic AEs rarely led to treatment discontinuation (TMC278: n=3 vs. EFV: n=9 patients). There were no fatal hepatic AEs.

## Conclusions

Overall, both TMC278 and EFV were well tolerated with no hepatic safety differences observed. Hepatic AEs were more common in co-infected than in HIV mono-infected patients (27% vs. 4%, respectively), but there were no differences between the two treatment groups. Virologic responses were similar for TMC278 and EFV within the co-infected and HIV mono-infected groups, and lower in co-infected than in HIV mono-infected patients.

